# Surgery

**Published:** 1907-03

**Authors:** E. W. Hey Groves

**Affiliations:** M.B., B.Sc. Lond., F.R.C.S.


### SURGERY.

**Bier's method of treating tubercular joints by the production of passive hyperæmia** can hardly be described as a new subject, inasmuch as it has found a place in current text-books for the last ten years. But owing to the fact that the treatment was long and tedious, it was never received with much enthusiasm in this country, and it probably never received the trial that it deserved. But during the past year Professor Bier has communicated to the German Surgical Congress, and also to the

*Münchener medicinische Wochenschrift*,[1] a very much extended scope for this treatment, viz. to all forms of acute inflammation, and already in Germany and America other surgeons who have tried it declare that it is of very great value.

During the past thirty years the attention of surgeons has been so much occupied with the elaboration of the principles discovered by Lord Lister, and of the operations that these antiseptic principles have made possible, that every other method of treatment has been regarded as of little importance. The prevention of infection and of suppuration has claimed so much attention, that the treatment of infected or inflamed tissues has been very little thought of. Of course, nothing can ever supersede the importance of preventing infection and suppuration of tissues, but, nevertheless, such infection will always occur, and it is a matter of great importance if any method can cut short the process and minimise its evil results. Now this is what is claimed for the method of artificial hyperæmia. As opposed to the old anti-phlogistic school, who regarded all inflammation as bad and hurtful, Bier's view is that inflammation is the natural cure of infection, and therefore should be encouraged. But although the principle underlying its application to tubercular and to inflammatory cases is no doubt the same, the method of its application is so different in the two classes of case that it will simplify matters if they are described separately.

In the case of tubercular joints, artificial congestion has been applied successfully[2] to all except the hip. A rubber bandage is wound round the limb at as great a distance from the joint as possible, and over a layer of gauze. In the case of the shoulder, the bandage is kept in place by calico strips round the neck and round the chest. The amount of constriction to be applied is a matter of great importance. The arterial supply as estimated by the pulse should not be altered, but the veins so compressed that the return of blood is hindered, and a congestion occurs below the bandage. The temperature of the part should be raised and not diminished. The bandage remains in its place for one to two hours daily, and the treatment of a case of moderate severity occupies from four to six months. If the bandage be left on too long in these cases acute inflammation is apt to supervene, and suppuration occur, and it is very important to bear this in mind in distinguishing between the treatment of tubercular and acute inflammatory cases.

It is not the primary object of this paper to deal with the tubercular cases, but in order to give completeness to the subject it may be well to briefly mention some recent publications which indicate the results of passive congestion as applied for these

[1] *München. med. Wchnschr.*, 1905, lii. 201, 263, 318; also Bier's "Hyperämie als Heilmittel," 1905.

[2] *Lancet*, 1905, i. 1091.

conditions. Habs[1] gives an account of 200 tubercular joint cases in which he used this method. Whilst all varieties of the disease may show improvement, yet he recognises certain well-marked differences in various groups of cases. Thus children react much better than adults. Mild chronic cases do better than acute ones. Cases of pure synovial disease, when there is no affection of the bones, are those in which the results of the congestive method are the best. The elbow, wrist, knee and ankle all give good results, but the shoulder has also been successfully dealt with. Now it is evident from these observations that Bier's method is of the greatest value in just those cases which would in any case be treated by expectant rather than operative measures. Therefore it is not so much a question of choosing between operation or passive hyperæmia, as of simply adding the congestive treatment to other methods of non-operative procedure. In the knee, however, although the cases may improve for a time, a large proportion ultimately require operation. In early cases of wrist and ankle disease the method is of particular value, because it is in these that mere immobilisation so often fails. In fact, Bier[2] recommends that in early cases the joints should not be kept at rest, but that passive movements, preferably carried out in a hot-water bath, should be regularly employed. Tubercular cases in which the skin is broken or in which there is any septic complication, are not suited for the treatment, for although artificial congestion is used both for septic and tubercular affections, it has to be applied in a different manner in each case, and therefore cannot be employed when the two conditions co-exist. Ullmann[2] reports three cases of tuberculous testes successfully treated by Bier's method. A rubber band was bound lightly round the base of the penis and scrotum for half to one hour daily. In these cases the tuberculous disease had infiltrated the skin, and in all of them the pain and discharge were lessened, and the tubercular infiltration became smaller and softer, whilst the patients gained in weight. One would like to know the further history of these cases. Polyak[3] has tried the method with most gratifying results for tuberculosis of the larynx. The rubber band is applied as low down in the neck as possible, but quite lightly so as not to cause any inconvenience to breathing. The relief of pain is said to be the first and most striking result, and is the safest criterion of the amount of good which is likely to be gained by persisting in the treatment.

But it is **the application of artificial congestion to acute inflammatory lesions of all kinds**, *e.g.* a whitlow, a carbuncle, osteomyelitis or suppurative mastoiditis, that constitutes the more recent chapter in the history of surgical progress. Bier himself

[1] *München. med. Wchnschr.*, 1903, l. 938; abstract in *Edinb. M. J.*, 1903, N.S., xiv. 359.

[2] *Lancet*, 1905, ii. 1737. [3] *Edinb. M. J.*, 1906, N.S., xx. 183.

began to apply his method in such cases as long ago as 1893, but it was only last year that his work was fully published, and his results corroborated by other observers.

In these cases the actual constricting agent is applied in the same way as above described, but it is allowed to remain in position for very much longer, that is to say for 20 to 22 hours out of the 24 For such a condition as a whitlow in the hand, the bandage is applied to the upper arm, for inflammation of the foot it is applied to the lower part of the thigh. Again, in striking contrast to the best conditions in tubercular cases is the fact that the more acute the inflammatory lesion is, the better result does the congestive treatment give. The pressure of the band should be very light, but soon after its application the limb becomes a fiery red, which spreads from the original focus right up to the bandage itself. Then, too, the whole becomes swollen and œdematous, and if any incisions exist they pour with serous exudation. The position of the bandage is changed every ten hours, and in the short intervals between the periods of constriction the limb is raised to favour absorption of the œdema.

As soon as the congestive reaction has set in the patient feels a great relief of pain, the pulse becomes slower and more regular, and the temperature falls. The temperature rises, however, between the periods of constriction, but this, which is marked at first, soon becomes much less, until in about three to six days a normal mean is maintained. If suppuration occurs the pus must be let out, but the incisions need not be so free as would ordinarily be the case. Of course, if septicæmia or pyæmia has already developed this treatment is of no avail, except in hastening the resolution of any particular focus. Cathcart[1] gives a summary of Bier's recent papers with some of his cases, and adds a number of his own personal observations at the Edinburgh Infirmary. The scope of the method is well shown by the following typical examples of large groups of cases.

*Suppression of commencing suppuration.*—On April 8th, 1904, the left wrist of a woman of 60, who had a septic wound of the breast, became inflamed, with a rigor and temperature rising to 103° F. Passive congestion applied for 20 hours daily cured the condition in three days, the pain, heat and raised temperature disappearing.

*Transformation of acute into cold abscess.*—This is a rare occurrence. A boy of 7 was admitted on July 28th with an acute abscess in the lower end of his thigh. An exploratory syringe drew off thick pus containing staphylococci. Under passive congestion the inflammation had gone by July 30th, and the raised temperature fell to normal. The abscess remained as a cold, fluctuating swelling. On August 5th a 1 c.m. long incision was made into it, and the pus pressed out without further opening. It healed by August 9th. This transformation of acute into

[1] *Scottish M. and S. J.*, 1906, xviii. 302.

chronic abscesses is not, however, to be waited for or expected as a routine. It is better to open abscesses at the time the congestion is first applied.

*Suppurating surfaces.*—In these the pus at first becomes more abundant, but thinner. Very shortly the pus ceases, sloughs are thrown off, and other parts recover which would have necrosed under ordinary treatment. And the suppurating process becomes definitely limited, instead of spreading. Inflamed joints and all kinds of arthritis have been successfully treated, but the more acute cases seem to show more brilliant results than the chronic ones, *e.g.* osteoarthritis. In joints containing pus only a diagnostic puncture is made, but the joint is neither opened nor drained. The part is kept at rest, but the joint is not immobilised, but, on the contrary, is subjected to gentle passive movement as soon as the passive congestion has produced a cessation of pain. A man of 18 was admitted seventeen days after a septic wound which had penetrated the left elbow-joint. The joint was red, swollen, painful, and fixed at a right angle, and pus could be squeezed plentifully from the wound. In three weeks' congestive treatment the joint had recovered and the fistula healed. Within another month he had a freely movable joint. A man aged 20 was admitted with acute suppuration in his right knee, the origin of which was unknown. All the typical signs of acute suppurative arthritis were present, including rigor and a high temperature. The syringe withdrew pus containing living staphylococci. After twelve days' treatment the temperature was normal, the swelling was less, the pain had ceased, and the patient could flex his knee to a right angle. The fluid in the knee was now turbid serum. He recovered the complete use of the knee-joint, so that he was taken as ward attendant at the clinic.

*Suppuration in tendon sheaths.*—In these cases it is important to make incisions directly pus is formed, but these need only be stab incisions, and no packing or draining is required. Pus pours from them readily enough when congestion is applied. A butcher of 43 had a wound of the little finger which had infected the tendon sheaths and cellular tissue up to the elbow with acute lymphangitis. Temperature was 102.1° F., and he was very ill. After four days' treatment all the local inflammation had gone, the temperature was normal, and after a few tendon sloughs had escaped the wounds healed, and he had good use in his hand except for a little stiffness of the finger. Out of 22 cases of tendon sheath suppuration, 14 recovered without sloughing. And the extraordinary and almost incredible rapidity of the cures are in marked contrast to the tedious course of such cases when treated by the old methods.

*Acute osteomyelitis.*—Bier has had 14 cases of acute osteomyelitis, of which 6 recovered with no necrosis, 5 with very little necrosis, 2 with extensive necrosis, and 1 died of pyæmia.

*Gonorrhœal arthritis.*—A married woman of 38, who had been infected with gonorrhœa some time previously, was attacked by typical acute arthritis of the right knee-joint. For a month rest, extension, fomentation and drugs were tried without avail, but within fifteen minutes of the application of the rubber bandage to the thigh the pain was easier, and in a few weeks she was able to walk about with a somewhat stiff knee.

Of course, in many cases disappointment follows the method, as in these it proves inefficacious, but if carefully applied to suitable cases it does not do harm, and with practice and experience its scope will greatly increase.

Besides the elastic bandage, various kinds of suction apparatus may be used, and Dr. Klapp, Professor Bier's assistant, has devised a number for use in different positions of the body. For example, in the case of acute mastoid disease a suction cup may be placed over the mastoid region, in addition to or instead of a band round the neck.[1] Bier states that if this is done, only a small puncture into the mastoid antrum is necessary, and the case quickly recovers with good functional results in 60 per cent. of his cases. Polyák[2] has used a suction cup for tonsillar and pharyngeal inflammation, but finds that a band low down in the neck will give great relief to such diverse conditions as nasal catarrh, pharyngitis, maxillary suppuration, or laryngitis. Rudolph[3] has devised a suction apparatus which can be applied to the cervix for inflammatory conditions of the uterus, and he reports very good results from its use.

Whilst the majority of observers are agreed as to the wonderful results obtained by passive congestion, yet there are some who have not had such good results. Bardenheuer, who is now a firm believer in the method, had very bad results from it until he was instructed in its method of application by one of Bier's assistants. Lexer[4] declares it to be merely "a game of chance" as to whether the inflammatory processes will be checked or spread by its use, and he states that he has found that it produces an extension rather than a limitation of suppuration in cases where the parts are unopened. But if incisions are made first, then even this observer admits the great value of the congestion. Stettiner[5] points out how suppurating cavities and sinuses with small mouths may be made to heal without enlargement by suction hyperæmia. This is of great value in treating abdominal fistulas or stitch abscesses. Sick[6] remarks, and other observers, including Bier himself, agree with him, that there are certain well-marked groups of cases in which the treatment should not be applied, because in them it is likely to do positive harm. These are:—Rapidly-spreading strepto-

[1] *Ann. Surg.*, 1906, xliv. 729. [2] *Loc. cit.*
[3] *Centralbl. f. Gynäk.*, 1905, xxix. 1185. [4] *Ann. Surg.*, 1906, xliv. 731.
[5] *Ibid.*, p. 734. [6] *Ibid.*, p. 730.

coccal infections, including erysipelas, inflammatory lesions in diabetes, varicose or congestive ulcers, and thrombo-phlebitis.

As to the mode of action of this treatment, we know little or nothing. The very rapid relief of pain, occurring generally in a few minutes to half an hour, must have a mechanical explanation, and Bier suggests that it is due to anæsthesia produced by the rapid œdema. The great effusion from the vessels of the part must flood the inflamed tissues with serum, leucocytes and opsonins, and it is possible that in this manner the invasion by micro-organisms is overcome. But in inflammation which results from chemical irritants the method is equally successful. It is of value in this connection to note the experiments of Perthes,[1] who injected strychnine solutions into animals' limbs, and found that by the use of a constricting band the supervension of toxic symptoms could be prevented, or so much delayed that the animal would survive a dose which would have killed it if it had been delivered into the free circulation. At any rate, it is impossible to rise from a perusal of the recent writings on this subject by so many different authors without feeling that a great addition has been made to our methods of treating common forms of disease. And it certainly involves the necessity of our making a personal study of the question, so that our patients may not be deprived of what may be a most valuable aid to recovery.

E. W. Hey Groves.

